# Combination of deep eutectic solvent and ionic liquid to improve biocatalytic reduction of 2-octanone with *Acetobacter pasteurianus* GIM1.158 cell

**DOI:** 10.1038/srep26158

**Published:** 2016-05-17

**Authors:** Pei Xu, Peng-Xuan Du, Min-Hua Zong, Ning Li, Wen-Yong Lou

**Affiliations:** 1 State Key Laboratory of Pulp and Paper Engineering, South China University of Technology, Guangzhou 510640, China; 2 Laboratory of Applied Biocatalysis, School of Food Science and Engineering, South China University of Technology, Guangzhou 510640, China

## Abstract

The efficient anti-Prelog asymmetric reduction of 2-octanone with *Acetobacter pasteurianus* GIM1.158 cells was successfully performed in a biphasic system consisting of deep eutectic solvent (DES) and water-immiscible ionic liquid (IL). Various DESs exerted different effects on the synthesis of (*R*)-2-octanol. Choline chloride/ethylene glycol (ChCl/EG) exhibited good biocompatibility and could moderately increase the cell membrane permeability thus leading to the better results. Adding ChCl/EG increased the optimal substrate concentration from 40 mM to 60 mM and the product *e.e.* kept above 99.9%. To further improve the reaction efficiency, water-immiscible ILs were introduced to the reaction system and an enhanced substrate concentration (1.5 M) was observed with C_4_MIM·PF_6_. Additionally, the cells manifested good operational stability in the reaction system. Thus, the efficient biocatalytic process with ChCl/EG and C_4_MIM·PF_6_ was promising for efficient synthesis of (*R*)-2-octanol.

Enantiomerically pure chiral alcohols are very useful and valuable chiral building blocks for the synthesis of pharmaceuticals, agrochemicals, liquid crystals and flavors^1^. Currently, optically chiral alcohols can be prepared by either chemical catalysts or biocatalysts, such as, free or immobilized enzymes and novel whole-cell biocatalysts^2^,^3^,^4^. Generally, whole-cell biocatalysts are preferable to isolated enzymes since they are benign enzymes suppliers with coenzyme regeneration *in situ*, no need for enzyme purification, mild reaction condition and less enzyme inactivation^5^.

Over the past decades, biocatalytic process have obtained great progress in the discovery of new biocatalysts as well as medium engineering, of which the development of new green solvent as a reaction medium is one of the key subjects in biocatalytic reactions. Ionic liquids (ILs) have been firstly studied for their potential in replacing current harsh organic solvents and have been applied to many biocatalytic processes and obtained numerous encouraging results^6^,^7^,^8^,^9^,^10^. However, the “green aspect” of these ILs is now largely contested and their possible toxicity and low biodegradability are recently reported in literatures^11^,^12^,^13^.

Deep eutectic solvents (DESs) have recently been developed as a new and attractive alternative to traditional ILs. Generally, DESs were eutectic mixtures of a quaternary ammonium salt and a metal salt or hydrogen bond donor (HBD) such as glycerol and urea^14^. DESs possess some unique merits including low cost, non-toxicity, easy preparation and better biocompatibility and biodegradability apart from those belonging to ILs (e.g. low melting temperature, low volatility and high themo-stability)^15^,^16^,^17^. Recently, DESs have gained much attention as promising reaction media for biocatalysis and biotransformation, and a number of enzymes like epoxide hydrolases, proteases and lipases, in many cases, have shown enhanced activity and stability in the presence of DESs based on choline or ethylammonium chloride paired with alcohols, acids or amides^18^,^19^,^20^,^21^. In addition, it was reported that DESs led to the structural change of horseradish peroxidase and consequently affected significantly its activity, giving the inspiring results^22^. To date, however, use of DESs as reaction medium for whole-cell biocatalysis has remained less explored^23^,^24^. Thereby, it is of great interest to investigate the effect of DESs on the biocatalytic reactions using microbial cells.

In the present work, diverse DESs were first tested for their potential as co-solvents for biocatalytic reduction of 2-octanone to (*R*)-2-octanol with *Acetobacter pasteurianus* GIM1.158 cells in DES-containing aqueous system (Fig. 1) and their biocompatibility and effects on the cell membrane integrity were studied simultaneously. Additionally, the effects of several crucial variables on the bioreduction of 2-octanone were investigated systematically. Then, water immiscible ILs were applied as performance additive to further improve the reaction efficiency since they were good substrate solubilisers and could improve the reaction efficiency to some degree. Furthermore, the reusability of the whole-cell biocatalyst in the biphasic system was explored.

## Results

### Comparison of the biocatalytic reduction of 2-octanone with *Acetobacter pasteurianus* GIM1.158 cells in different DES-containing systems

Up to now, there is still few reports on biocatalytic reduction of ketones using microbial cells in DES-containing reaction systems. Therefore, we initially performed asymmetric reduction of 2-octanone to (*R*)-2-octanol, catalyzed by *A. pasteurianus* GIM1.158 cells, in various DES (Table 1) -containing systems in order to focus on the influence of DESs on the bioreduction.

It was noted that the *Acetobacter pasteurianus* GIM1.158 cells were capable of catalyzing the asymmetric reduction of 2-octanone in various DES-containing systems with a high product *e.e.* of above 98.5%, while the biocatalytic reaction varied greatly with the use of different DESs with respects to the initial reaction rate and maximum yield at a reaction time of 2 h (Table 2). The addition of ChCl/OA, ChCl/MA into aqueous buffer system led to absolute inhibition of *A. pasteurianus* GIM1.158 cells. Notably, there was a slight but clear increase in initial reaction rate (from 1.37 *μ*molmin^−1^ to 1.61 *μ*molmin^−1^) and maximum yield (from 80.1% to 85.5%) when the DES ChCl/EG was added into aqueous buffer system at a volume ratio of 10%. Of the seven DESs that were tested, ChCl/EG gave the fastest initial reaction rate and the highest maximum yield, and consequently was considered as the most suitable DES for the bioreduction reaction.

### Biocompatibility of various DESs with *Acetobacter pasteurianus* GIM1.158 cells

To understand the effect of DESs on the bioreduction reaction, the biocompatibility of the DESs were investigated in-depth by using the sugar metabolic activity retention (MAR) of the microbial cell. MAR depends on the cells tolerance to solvents and is an easy indicator of cell viability after 24 h exposure to co-solvent systems consisting of various DESs, in the absence and presence of substrate.

**Figure 2:**
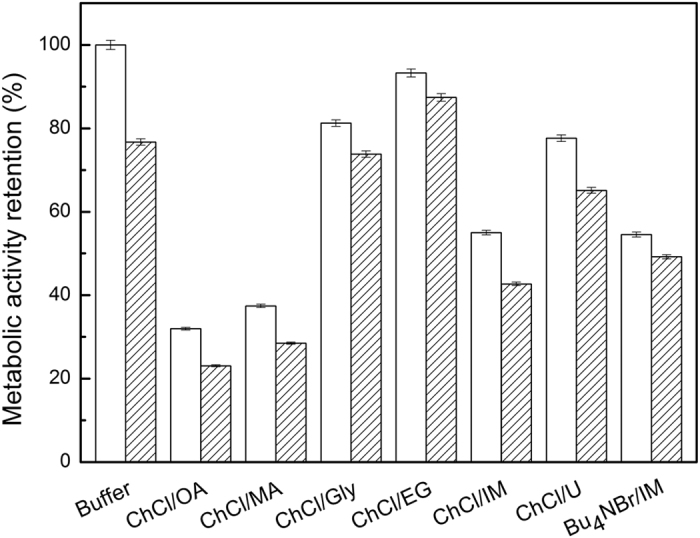
The sugar metabolism activity retention of *Acetobacter pasteurianus* GIM1.158 cells in various DES -containing co-solvent systems without (white) and with (pattern) substrate.

As illustrated in Fig. 2, the MAR values of the cells in all the tested DES-containing systems were lower than that in DES-free buffer in the absence of 2-octanone, indicating that the tested DESs exhibited different levels of toxicity to *A. pasteurianus* GIM1.158 cells. The MAR values in diverse DES-containing systems varied greatly with different DESs. For example, ChCl/EG exhibited the best biocompatibility with the cells, giving the highest MAR value of 92%, which was in good agreement with the observation of the catalytic efficiency in terms of the maximum yield and the initial reaction rate in the reaction system (Table 2). When the HBDs were OA and MA, the MAR values decreased greatly to only 30% and 35%, respectively. Also, it was obvious that in the presence of 2-octanone (40 mM), the MAR value of the cells after 24 h incubation decreased in all the systems as compared with those without substrate, possibly due to the toxicity of the substrate to the cells. Interestingly, the MAR value with substrate was reduced only by 5% compared to that without substrate in the ChCl/EG-containing system, while the counterpart value was up to 24% in the aqueous buffer.

### Effect of various DESs on cell membrane permeability

It is possible that DESs used in this work might affect the mass transfer of substrate and product across the cell member and hence influence the bioreduction reaction. The increases in medium OD_260_ and OD_280_ values, the indicator of the release of intracellular components (presumably nucleic acids and proteins) into the medium during 24 h incubation with different DESs (10%, v/v) were measured. The increases in OD were taken as a direct measure of the DESs’ effect on the cell membrane permeability. Subsequently, flow cytometer (FCM) with propidium iodide (PI) as cell fluorescein dye was used to detect the cell membrane integrity.

As can be seen from Table 3, addition of various DESs into aqueous buffer system (as the control) could increase the cell membrane permeability of *Acetobacter pasteurianus* GIM1.158 cells. It was obvious that the OD_260_ and OD_280_ values were higher in DES-containing systems than in the aqueous control system. ChCl/EG gave relatively low OD_260_ and OD_280_ values, indicating that ChCl/EG could befittingly increase the cell membrane permeability and thus effectively reduce the product inhibition. However, when the HBDs were OA and MA, the cells membrane were destroyed seriously, in line with the poor catalytic efficiency in the biocatalytic reduction of 2-octanone to (*R*)-2-octanol (Table 2). Of all the DESs used, the lowest OD_260_ and OD_280_ values were observed in the ChCl/EG-containing system with the best biocatalysis result and biocompatibility. The results described above were further reinforced by the FCM data (see Supplementary Fig. S1). Relatively higher cell membrane integrity of 87.6% was maintained in the presence of ChCl/EG, whereas ChCl/MA caused a serious damage of the membrane integrity from 94.9% to 2.5% compared with that in the aqueous buffer.

### Optimization of biocatalytic production of (*R*)-2-octanol in ChCl/EG-containing system

In order to further improve the reaction efficiency with respect to the initial reaction rate and the yield, a systematic investigation was made of the effects of several key variables such as DES content, substrate concentration, buffer pH and reaction temperature on the reaction.

**Figure 3:**
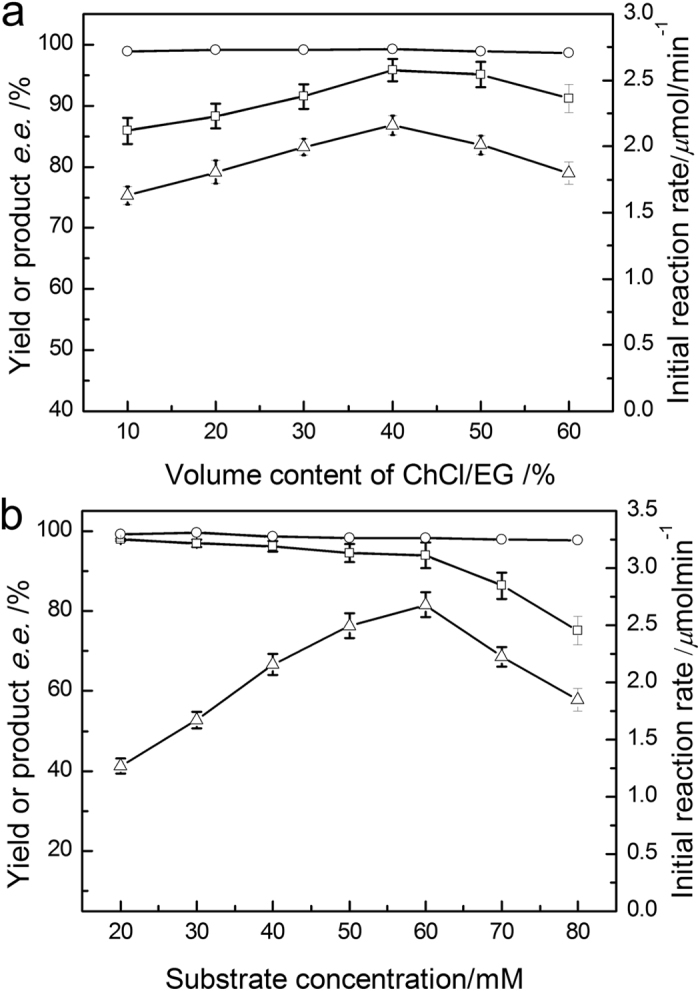
Effect of ChCl/EG (1:2) content (**a**) and substrate concentration (**b**) on the product yield (□), product *e.e.* (○) and initial reaction rate (△) of the biosynthesis of (*R*)-2-octanol with the biocatalyst in monophasic system. Reaction conditions: (**a**) TEA-HCl buffer (50 mM, pH 5.0, 10.0 mL) containing different content of ChCl/EG (v/v), 2-octanone (40 mM), isopropanol (500 mM), *Acetobacter pasteurianus* GIM1.158 cell (25 mg mL^−1^), 35 °C, 180 rpm; (**b**) TEA-HCl buffer (50 mM, pH 5.0, 10.0 mL) containing of ChCl/EG (40%, v/v), different concentration of 2-octanone, isopropanol (500 mM), *Acetobacter pasteurianus* GIM1.158 cell (25 mg mL^−1^), 35 °C, 180 rpm.

We initially investigated the effect of DES content on the biocatalytic process, since some previous studies have reported that the concentration of hydrophilic ILs markedly affected the activity of whole-cell biocatalysts. As shown in Fig. 3a, an increase in ChCl/EG content up to 40% brought about a clear increase in the initial reaction rate and the yield, while further increase in DES concentration led to a decline in the reaction efficiency. The product *e.e.* was consistently above 98.5% over the range.

It is well known that buffer pH plays a crucial role in biotransformation processes. As depicted in Supplementary Fig. S2, the buffer pH significantly affected the bioreduction of 2-octanone in the ChCl/EG-containing system and the optimal pH for the reaction was pH 5.0. In addition, Supplementary Fig. S3 illustrates that 35 °C was considered as the optimum temperature for the bioreaction. Our previous study indicated that isopropanol was the optimal co-substrate for the bioreduction of 2-octanone. Supplementary Fig. S4 shows that the co-substrate concentration was closely related to the initial reaction rate and product yield and the preferable concentration of isopropanol was 500 mM.

As evident in Fig. 3b, the reaction accelerated markedly with increasing substrate concentration from 20 mM to 60 mM. However, the product yield decreased in this range while being above 90% and a remarkable decline was observed with a higher substrate concentration. Thus, the optimal substrate concentration was 60 mM in the DES-containing system, which was 3 times higher than that in aqueous system. Under the optimized conditions, the maximum product yield, the reaction in initial rate and product e.e. were 95.7%, 2.60 μmolmin^−1^ and 98.9%, respectively.

### The biocatalytic reduction of 2-octanone in DES-buffer/IL biphasic system

The ChCl/EG-containing system described above brought indeed a remarkable improvement in the reaction efficiency in respects of substrate loading and the maximum product yield. The serious product and substrate inhibition, however, still existed. In an attempt to further improve the substrate loading significantly, the traditional hydrophobic ILs were introduced, as a substrate reservoir and *in situ* extractant of toxic products, to the DES-containing monophasic system.

Of the five tested hydrophobic ILs, the best biocompatibility was obtained by C_4_MIM·PF_6_ regardless of the presence of 2-octanone (see Supplementary Fig. S5). Besides, the hydrophobic ILs narrowed the difference of MAR values of the cells with and without 2-octanone, indicating that the toxicity of 2-octanone to the cells was weakened by the ILs. Though with relatively low product yield and initial reaction rate, C_4_MIM·PF_6_ manifested apparently positive effect on the reaction in comparison with the other ILs (Table 4). In cases of anion being either PF_6_^−^ or Tf_2_N^−^, both the initial reaction rate and product yield decreased with the elongating of the alkyl chain of cation. Supplementary Table S1 displayed that both the substrate and product preferably distributed in the C_4_MIM·PF_6_ with higher partition coefficients, which accounted for the good performance of the cells in the C_4_MIM·PF_6_-containing biphasic system. Thus C_4_MIM·PF_6_ was selected as the second phase in the following experiments.

**Figure 4:**
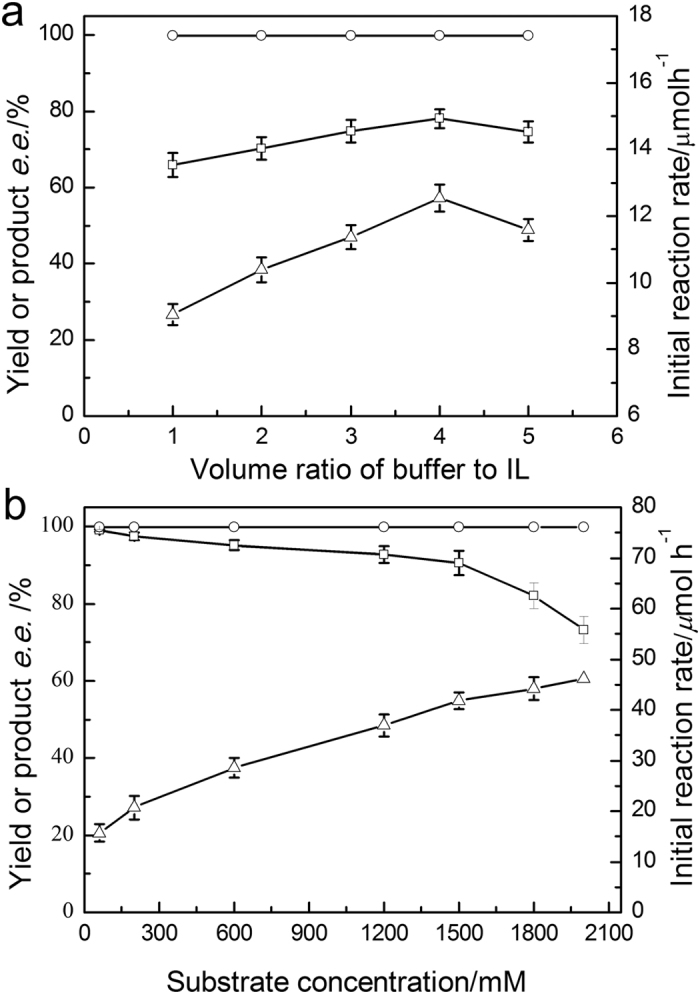
Effect of V_aq_/V_IL_ ratio (**a**) and substrate concentration (**b**) on the product yield (□), product *e.e.* (○) and initial reaction rate (△) of the biosynthesis of (*R*)-2-octanol with the biocatalyst in biphasic system. Reaction conditions: (a) 2-octanone (60 mM), C_4_MIM·PF_6_ (1.0 mL), different volume of TEA-HCl buffer (50 mM, pH 5.0) containing ChCl/EG (40% v/v), isopropanol (500 mM), *Acetobacter pasteurianus* GIM1.158 cell (25 mg mL^−1^), 35 °C, 180 rpm; (**b**) different concentration of 2-octanone, C_4_MIM·PF_6_ (1.0 mL), TEA-HCl buffer (50 mM, pH 5.0, 4.0 mL) containing ChCl/EG (40%, v/v), isopropanol (3 M), *Acetobacter pasteurianus* GIM1.158 cell (25 mg mL^−1^), 35 °C, 180 rpm.

Through optimizing the volume ratio of DES/buffer to IL, buffer pH, reaction temperature and co-substrate concentration, the product yield was elevated to 90% approximately (see Fig. 4a and Supplementary Figs S6–S8). Importantly, in the ChCl/EG-buffer/C_4_MIM·PF_6_ biphasic system, the optimum substrate concentration was enhanced up to 1.5 M (Fig. 4b), 25-fold higher than that in the ChCl/EG-buffer system. Unexpectedly, the product yield decreased with the increasing of substrate concentration.

### Operational stability of *Acetobacter pasteurianus* GIM1.158 cells in various systems

The relative activities of the cells employed for the first batch in the bioreduction of 2-octanone were defined as 100%. After recycling operating for nine batches, the relative activities of the cells in C_4_MIMPF_6_-containing biphasic system and ChCl/EG-buffer system retained above 60%, while the counterpart activity in TEA-buffer system was below 40% (see Supplementary Fig. S9). Besides, the cells still kept 65.3% of their original activity after being reused for ten batches in the ChCl/EG-buffer/C_4_MIMPF_6_ biphasic system, which indicated that the hydrophobic C_4_MIMPF_6_ could extract the substrate and product effectively to reduce their toxicity to the cells.

## Discussion

Novel reaction medium is one of the crucial means to enhance the reaction efficiency and DESs have shown great potential in the biocatalytic reaction as co-solvents. In this work, DES, ChCl/EG, manifested good biocompatibility with the *Acetobacter pasteurianus* GIM1.158 cells and the synthesis of (*R*)-2-octanol can be successfully conducted with high yield and excellent product *e.e.*, by means of the biocatalytic anti-Prelog stereoselective reduction of 2-octanone using the cells in ChCl/EG -containing system.

The addition of ChCl/EG to the reaction system could effectively lower the toxicity of the substrate to the cells as illustrated by the MAR decrease between the cells incubated with and without 2-octanone. The relatively low MAR values of the DESs with HBDs being OA and MA correlated to the poor performance of the cells in these two DES-containing systems, implying their high toxicity to the cells. The relatively high toxicity of acid-HBD DESs to microorganism has been proved in the work reported by Zhao *et al*.^25^. Moreover, it was populated that the addition of acid HBD could seriously change the pH of the aqueous system, thus affecting the cells activity. Unexpectedly, the pH value just had a variation of 0.15 by adding ChCl/EG up to 60% (v/v) (see Supplementary Table S2), suggesting that the pH change was not the main reason why the DES could boost the bioreduction reaction. Up to now, a few literatures have also been reported about the toxicity of DESs. For example, Hayyan *et al*.^26^,^27^ found that four ChCl-based DESs exerted extremely low toxicity to all the studied bacteria including *B. subtilis, S. aureus, E. coli*, and *P. aeruginosa.* The toxicity variance of the tested DESs mainly depends on the structure difference of the HBDs, which directly affect the hydrogen bond formation between the two components. The toxicity indirectly reflects the biocompatibility of DESs and puts an influence on their application in whole-cell biocatalysis. The relatively lower viscosity of ChCl/EG compared to the other DESs^25^,^28^,^29^, could be beneficial for the mass transfer of the substrate and product, thus also accounting for the good biocatalytic efficiency of the biocatalyst in the ChCl/EG-containing system. The viscosity of these binary eutectic mixtures is essentially governed by the hydrogen bonds, van der Waals, electrostatic interactions, temperature as well as the ratio of the two components.

As well known, there is close relation between the cell activity and the cell membrane integrity^30^. In our previous studies, it was found that diverse ILs and DESs had very different effect on the permeability and integrity on the cell membrane^31^,^32^. A moderate enhancement of the membrane permeability could improve the reaction efficiency^33^. It seemed that the mass transfer of the substrate and product turned to be easier for the slight-damaged cells. The present work has proved that ChCl/EG is the most suitable co-solvent for the biocatalytic production of (*R*)-2-octanol, because of its good biocompatibility with the cells. On the other hand, in spite of moderately increasing the cell membrane permeability to facilitate the mass transfer, it is possible that the ChCl/EG could allow the enzymes capable of catalyzing the bioreduction reaction to come out of the cells. In order to further figure out whether the reduced catalytic activity of the cells in different DESs could be attributed to the cell membrane rupture, the activities of both the cell debris and the cell extract after the cells being lysed by ultrasonic was qualitatively determined firstly. As depicted in the Supplementary Table S3, only the cell extract with NADH was capable of catalyzing the bioreduction reaction, indicating that the corresponding reductase was an intracellular enzyme. Additionally, it should be noted that the coenzyme was necessary for the bioreduction process *in vitro*. At the same time, the supernatants were assayed after removal of the cell pretreated with different DESs (10, v/v) for 2 h, which was the same as the reaction time in Table 2, but no significant activity toward 2-octanone was observed. It could be concluded that the cell membrane integrity is indeed closely related to the cells activity, and the biocompatible ChCl/EG didn’t lead to remarkable leak of the enzymes capable of catalyzing the bioreduction reaction in the microbial cell to the reaction medium during the reaction process. Also, it can be speculated that the DESs could cause the cell membrane expansion, which facilitated the substrate and the product to come in and out of the cells, rather than lyse the cells resulting in the cell death. However, serious increase of the permeability made it hard for the cells to maintain their catalytic activity. This proved that a moderate increase of the cell membrane permeability was beneficial for increasing the reaction efficiency. The ILs’ ability to stabilize the enzyme has been reported^34^ and several literatures about the effect of DESs on free enzyme activity have been also published^18^,^19^,^20^. Thus, it will be of great interest to further understand the mechanism of how DESs affect the cell activity in different aspects. There are no certain interpretations to illustrate clearly how the solvent could affect the catalytic performance of a certain microbial cell. In fact, the reason for the greatly different biocatalytic reaction using various DESs may lie in many different aspects, not just the cell membrane permeability. The enzyme inhibition, protein denaturation or modification of the cell membrane by the solvent could also cause a molecular toxicity to a given microorganism^35^. The related study is ongoing in our laboratory.

After optimizing the reaction conditions, the initial reaction rate and the maximum yield improved markedly compared to those in buffer (2.60 μmolmin^−1^ vs 1.37 μmolmin^−1^, 95.7% vs 80.1%). The optimum substrate concentration also had a significant increase from 40 mM to 60 mM, which still couldn’t meet the demand in industrial exploitation. In addition, the whole-cell still maintained high activity at a reaction medium consisting of 60% (v/v) ChCl/EG, which also implied their better biocompatibility to the microorganism.

Traditionally, hydrophobic ILs have been used as the performance additive to obtain a high substrate loading. Herein, such an obviously better catalytic efficiency was observed in the C_4_MIM·PF_6_-containing biphasic system in the Table 4. The difference of the catalytic efficiency in the five IL-containing system may be attributed to the following reasons: (a) the biocompatibility of the ILs to the cells decreases with the elongating of the alkyl chain of cation, resulting in the drop of the cells activity; (b) the longer of the alkyl chain, the more viscous of the IL and the greater of the mass transfer resistance; (c) the solubility difference of the substrate exists in different ILs. Based on the speculation described above, the water-immiscible C_4_MIM·PF_6_ was found to be more biocompatible with the *Acetobacter pasteurianus* GIM1.158 cells, which was in consistent with another report^36^. The relation between the toxicity (biocompatibity) of imidazole-based ILs and their alkyl chain length has been discussed in many papers^6^,^37^,^38^. Besides, The relatively low viscosity of C_4_MIM·PF_6_ may also contribute to its good performance^39^. High viscosity of the IL will hinder the diffusion rate of the 2-octanone from the IL phase to the aqueous phase, then directly affecting the its utilization efficiency by the cell. This phenomenon has been already observed in the situation of various DESs. It has been proved that the viscosity of ILs increased as the alkyl chain of the IL chain elongated (i.e., increasing *n* value), either for C_n_MIM·PF_6_ or C_n_MIM·Tf_2_N^40^. When various anions (PF_6_^−^or Tf_2_N^−^) were attached to the imidazolium cation, the initial reaction rate and the maximum yield were greatly different, indicating the anion exerted a substantial influence on the catalytic performance. Furthermore, because of the higher partition coefficients of those two compounds in the IL phase, permanent cell viability can benefit from the lower concentration of 2-octanone or the (*R*)-2-octanol in the aqueous phase, in which the microbial cells mainly remain. The toxic or inhibitory effect of the substrate/product can also be alleviated. However, due to the limit of the concentration of substrate available for the whole-cell biocatalysis, the initial reaction rate decreased clearly in the biphasic system (Table 4). Overall, the initial reaction rate and the maximum yield were tightly associated with the partition coefficients of the substrate 2-octanone in the IL/aqueous system. Therefore, the ILs affected the reaction efficiency mainly via influencing the substrate concentration in the aqueous phase of the biphasic system.

For a better understanding of the bioreduction performed in the DES/IL biphasic systems, the effects of several crucial variables were also studied. It has been reported that enzymes and active cells are inactivated by direct contact with the interface between the aqueous and the non-aqueous phases^41^,^42^. A higher V_aq_/V_IL_ ratio (above 4/1, Fig. 4a) possibly led to a higher concentration of substrate in hydrophobic phase and lower substrate availability by the cells. In the biphasic system, the optimal concentration of the co-substrate, isopropanol, was far higher than that in the aqueous system and isopropanol may act as a permeabilizer to enhance the cell membrane permeability moderately. Similarly, the optimal substrate concentration increased up to 1.5 M (Fig. 4b), possibly because of the great solubility of the substrate and product in C_4_MIM·PF_6_ thus lowering their inhibitory and toxic effect to the cells. Besides, the DES, ChCl/EG, could allow the cells to tolerate relatively high substrate concentration compared with those in neat aqueous buffer without ChCl/EG. To our knowledge, the maximum substrate concentration and productivity of biotransformation of 2-octanone to (*R*)-2-octanol in present work is far better than the other reports^4^,^43^. Finally, the whole-cell biocatalyst manifested much better operational stability in the ChCl/EG-buffer/C_4_MIM·PF_6_ system compared to those in systems with and without ChCl/EG. During the first five batches, the relative activity of the cell after each batch of re-use decreased no significantly in the biphasic system (24 h per batch), indicating its low toxicity to the *Acetobacter pasteurianus* GIM1.158 cells. After recycled for 10 batches, the reaction mixture was determined after removal of the cells and the product and showed no significant activity toward 2-octanone, implying that a very little amount of or even no the enzymes responsible for the bioreduction reaction were contained in the reaction medium. It could reach the same conclusion that the ChCl/EG had benign biocompatibility with the *Acetobacter pasteurianus* GIM1.158 cells and didn’t cause remarkable leak of the related redox enzymes during each batch (2 h per batch for the aqueous system and 16 h per batch for the biphasic system, respectively). The reason for the good reusablity in the biphasic systme lies not only in the excellent solvent properties of the IL C_4_MIM·PF_6_ for the toxic substrate and product, but also in the benign biocompatibility of the ChC/EG and C_4_MIM·PF_6._

In conclusion, this work offers an approach for the synthesis of (*R*)-2-octanol catalyzed by *Acetobacter pasteurianus* GIM1.158 cells in a biphasic system consisting of ChCl/EG and C_4_MIM·PF_6_, which shared the advantages of good biocompatibility with the cells, and acting as a good substrate reservoir. The DES ChCl/EG could moderately increase the cell membrane permeability and improve the reaction efficiency. The high substrate concentration (1.5 M), as well as the excellent operational stability of the whole-cell were obtained in the ChCl/EG-buffer/C_4_MIM·PF_6_ biphasic system. These results open the way to use DESs and *Acetobacter pasteurianus* GIM1.158 cells for efficient synthesis of (*R*)-2-octanol in practical application.

## Methods

### Biological and chemical materials

*Acetobacter pasteurianus* GIM1.158 was purchased from Guangdong Culture Collection Center. 2-Octanone (99% purity) and ethyl acetoacetate were purchased from Alfa Aesar (USA). (*R*)-2-Octanol (98% purity) and (*S*)-2-octanol (98% purity) were from Sigma-Aldrich (USA). The five ILs used in this work, shown in Table 1, were purchased from Lanzhou Institute of Chemical Physics (China). All other chemicals were from commercial sources and were of analytical grade.

### Preparation of DESs

The preparation of various DESs was according to our previous report^32^ and the abbreviations of all the DESs could be found in Table 1. Briefly, ChCl and a hydrogen-bond donor were added at a molar ratio of 1:2 (3:7 for tetrabutylammonium bromide and imidazole) into a beaker and mixed with constant stirring at 100 °C for 1–2 h until a clear liquid was formed. The DESs-containing aqueous solution was prepared by dissolving a certain DES TEA-HCl buffer (50 mM, pH 5.0) at an appropriate concentration (v:v).

### Cell cultivation

*Acetobacter pasteurianus* GIM1.158 cells were cultivated according the previous report^44^. The cells were separated from the cultivation broth at 8000 rpm for 10 min and stored for further use.

### General procedure for biocatalytic asymmetric reduction of 2-Octanone

In a typical experiment, DES-buffer (10% of DES in TEA-HCl, 50 mM, pH 5.0, 10.0 mL) containing wet cells (25 mg mL^−1^) and co-substrate (500 mM) was added to a Erlenmeyer flask capped with a septum, and pre-incubated in a water-bath shaker at 120 rpm and an appropriate temperature for 15 min. The reaction was initiated by adding a fixed amount of 2-octanone to the mixture. Aliquots (50 μL) were withdrawn at specified time intervals. The product and the residual substrate were extracted with ethyl acetate (100 μL) for twice containing *n*-decane (10.0 mM, as internal standard) prior to GC analysis. For the bioreduction performed in the DES-containing buffer/IL biphasic system, the volume ratio of DES-containing aqueous buffer to IL was 4:1. Details about substrate concentration, co-substrate concentration, buffer pH and reaction temperature are specified for each case.

### Cell metabolic activity retention measurement

The metabolic activity retention (MAR, %) of *Acetobacter pasteurianus* GIM1.158 cells was defined as the ratio of the consumed glucose amount by the cells pretreated in various DES/IL-containing media to that by the cells pretreated in aqueous buffer. The MAR of the cells was assayed after 24 h exposure to various DESs (10%, v/v)-TEA-HCl buffer (50 mM, pH 5.0) co-solvent systems in the presence and absence of 2-octanone (40 mM), respectively, in a rotary incubator set at 30 °C and 180 rpm. After separated from the reaction medium, the cells were transferred to glucose solution (10 mL, 10 g L^−1^), and then incubated at 30 °C and 180 rpm for 4 h. The glucose concentration in the medium was then determined by HPLC. The HPLC system (Waters 996 pump, Waters Corp., USA) was equipped with a Bio-Rad Aminex HPX-87H column (7.8× 300 mm) and a refractive index detector (Waters 2410, Waters Corp., USA).

### Cell membrane permeability assay

In a typical experiment, different DESs (10%, v/v) co-solvent mixtures (20 ml) or aqueous Tris-HCl buffer (50 mM, pH 5.0) containing *Acetobacter pasteurianus* GIM1.158 cells (25 mg mL^−1^) were incubated in a Erlenmeyer flask at 30 °C and 180 rpm. The cell-free supernatants (2.0 ml) containing DESs and released intracellular components were withdrawn from the co-solvent system at 0 h (controls with DESs) and 24 h. The OD_260_ and OD_280_ values of the samples were determined using ultraviolet spectrophotometer (Shimazu UV-2550). The OD_260_ and OD_280_ values at 24 h were corrected for the absorbance of the DESs by subtracting the corresponding 0 h absorbance values, hence these corrected values for the 24 h samples were a measure of the release of intracellular components (primarily nucleic acids and proteins) into the medium during incubation with DESs.

For the flow cytometry (FCM) assay, the cell was pretreated with various DESs according to our previous report^32^. After that, the cell suspension was diluted to be 10^6^ cfumL^−1^ and dyed with propidium iodide (a membrane impermeable fluorescent nucleic acid stain) at 4 °C in the dark, and then was subject to a BD FACS Verse Coulter to determine the cell membrane integrity. The fluorescent emission was excited at 535 nm, and recorded at 550–600 nm. Data from FCM were analyzed using BD FACSuite software.

### Determination of partition coefficients

The partition coefficients were determined by dissolving 2-octanone or (*R*)-2-octanol (60 mM) in each biphasic system consisting of IL and buffer (1:1, v/v) containing ChCl/EG (40%, v/v). Aliquots (20 μL) were sampled and extracted with ethyl acetate (100 μL) containing *n*-decane (10.0 mM, as internal standard) after shaking at 220 rpm and 35 °C for 24 h. The concentrations of 2-octanone and (*R*)-2-octanol in both phases were then analyzed by GC.

### Operational stability of the cells

ChCl/EG-buffer (40% in TEA-HCl, 50 mM, pH 5.0, 4.0 mL) containing wet cells (25 mg ml^−1^) and co-substrate (3 M) was mixed with C_4_MIM·PF_6_ (1 mL) containing 1.5 M 2-octanone in a Erlenmeyer flask capped with a septum. Then the reaction was performed in a shaker at 180 rpm and 35 °C and repeated for ten batches (24 h per batch). For each batch, the cells were separated, washed two times with distilled water and added again to a fresh reaction medium for next batch. Aliquots (10 μL) from the aqueous and non-aqueous phase were withdrawn at specified time. The product and the residual substrate were extracted with ethyl acetate (50 μL) for twice containing *n*-decane (10.0 mM, as internal standard) prior to GC analysis. The relative activity of the cells employed for the first batch was defined as 100%. In the aqueous system containing DES or not, the reaction was conducted referring to the optimal conditions for certain time (per batch).

### GC analysis

The reaction mixture was analyzed by a Shimadzu GC-2010 with a flame ionization detector and a HP-chiral CB column (30 m × 25 mm × 0.25 μm) (USA). The split ratio was 50:1. The injector and the detector were both at 250 °C. The carrier gas was nitrogen (>99.9). 2-Octanol was derived with trifluoroacetic anhydride before GC analysis. The column temperature was held at 110 °C and the flow rate of nitrogen was 0.75 mL/min. The retention times for derived 2-octanol, *n*-decane and 2-octanone were 3.9, 4.1 and 4.7 min. For the determination of the product *e.e.*, the column temperature was kept at 85 °C for 15 min while the flow rate of nitrogen in the column was 0.5 mLmin^−1^. The retention times for (*S*)-2-octanol and (*R*)-2-octanol were 13.06 and 12.79 min, respectively. The average error for this determination was <1.0%.

## Additional Information

**How to cite this article**: Xu, P. *et al*. Combination of deep eutectic solvent and ionic liquid to improve biocatalytic reduction of 2-octanone with *Acetobacter pasteurianus* GIM1.158 cell. *Sci. Rep.* **6**, 26158; doi: 10.1038/srep26158 (2016).

## Supplementary Material

Supplementary Information

## Figures and Tables

**Figure 1:**
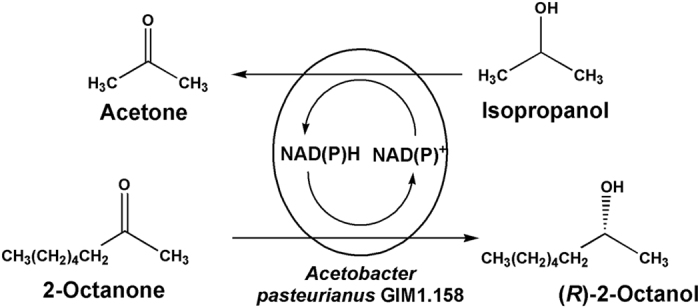


**Table 1:** Abbreviations of ILs and the components of DESs used in this work.

**Name**	**Abbreviation**
Choline chloride	ChCl
Urea	U
Glycerol	Gly
Ethylene glycol	EG
Oxalic acid	OA
Malonic acid	MA
Imicazole	IM
tetrabutylammonium bromide	Bu_4_NBr
1-butyl-3-methylimidazolium hexafluorophosphate	C_4_MIM·PF_6_
1-pentyl-3-methylimidazolium hexafluorophosphate	C_5_MIM·PF_6_
1-ethyl-3-methylimidazolium bis(trifluoromethanesulfonyl)imide	C_2_MIM·Tf_2_N
1-butyl-3-methylimidazolium bis(trifluoromethanesulfonyl)imide	C_4_MIM·Tf_2_N
N-butyl-N-methylpiperidinium bis(trifluoromethanesulfonyl)imide	PP_14_·Tf_2_N

**Table 2:** The biocatalytic asymmetric reduction of 2-octanone to (*R*)-2-octanol with *Acetobacter pasteurianus* GIM1.158 cells in various DES-containing systems^a^.

**DES**	**Initial reaction rate (*****μ*****molmin**^**−1**^)	**Reaction time (h)**	**Yield**^b^ **(%)**	***e.e.*** **(%)**
Buffer	1.37 ± 0.012	2.0	80.1 ± 0.71	98.5 ± 0.76
ChCl/OA(1:2)	n.d.	2.0	n.d.	n.d.
ChCl/MA(1:2)	n.d.	2.0	n.d.	n.d.
ChCl/Gly(1:2)	1.14 ± 0.011	2.0	75.2 ± 0.63	98.7 ± 0.91
ChCl/EG(1:2)	1.61 ± 0.014	2.0	85.5 ± 0.59	98.8 ± 0.87
ChCl/IM(1:2)	1.08 ± 0.011	2.0	69.3 ± 0.61	99.0 ± 0.98
ChCl/U(1:2)	1.17 ± 0.008	2.0	70.1 ± 0.69	98.6 ± 0.68
Bu_4_NBr/IM(3:7)	0.97 ± 0.009	2.0	62.6 ± 0.56	98.9 ± 0.75

TEA-HCl buffer (10.0 mL, 50 mM, pH 5.0) containing various DESs (10%, v/v), 2-octanone (40 mM), 35 °C, wet cells (25 mg ml^−1^), isopropanol (500 mM), 180 rpm.

^a^Reaction conditions:

^b^The maxmium yield.

**Table 3:** Effect of DESs on the cell permeability of *Acetobacter pasteurianus* GIM1.158 cells.

**DES**	**OD**_**260**_^a^	**OD**_**280**_^a^
Buffer	0.015	0.029
ChCl/OA(1:2)	0.126	0.292
ChCl/MA(1:2)	0.113.	0.258
ChCl/EG(1:2)	0.037	0.061
ChCl/Gly(1:2)	0.071	0.118
ChCl/IM(1:2)	0.062	0.121
ChCl/U(1:2)	0.085	0.114
Bu_4_NBr/IM (3:7)	0.077	0.131

^a^OD_260_ and OD_280_ values were the increases in OD_260_ and OD_280_ of the medium from 0 h to 24 h,and thus indicate the cellular contents released during that period, which were taken as a measure of cell membrane permeability.

**Table 4:** Effect of various ILs on the biocatalytic asymmetric reduction of 2-octanone by *Acetobacter pasteurianus* GIM1.158cells^a^.

**IL**	**Initial reaction rate (*****μ*****molh**^**−1**^)	**Reaction time (h)**	**Yield**^b^ **(%)**	***e.e.*****(%)**
C_4_MIM·PF_6_/buffer	9.17 ± 0.086	16	68.3 ± 0.66	>99.9
C_5_MIM·PF_6_/buffer	7.52 ± 0.069	16	59.1 ± 0.49	>99.9
C_2_MIM·Tf_2_N/buffer	6.94 ± 0.068	24	55.9 ± 0.52	>99.9
C_4_MIM·Tf_2_N/buffer	5.27 ± 0.046	24	45.3 ± 0.38	>99.9
PP_14_·Tf_2_N/buffer	3.16 ± 0.027	24	35.6 ± 0.35	>99.9

Diverse IL (1.0 mL), TEA-HCl buffer (1.0 mL, 50 mM, pH 5.0) containing ChCl/EG (40%, v/v), 2-octanone (60 mM), wet cells (25 mg mL^−1^), isopropanol (500 mM), 35 °C, 180 rpm.

^a^Reaction conditions.

^b^The maxmium yield.
