# Supplementary material for: Combination of deep eutectic solvent and ionic liquid to improve biocatalytic reduction of 2-octanone with Acetobacter pasteurianus GIM1.158 cell
Source: Sci Rep. 2016 May 17;6:26158. doi: 10.1038/srep26158 (PMC4868980; doi:10.1038/srep26158)
Supplement: Supplementary Information [file srep26158-s1.pdf]

Supplementary Information

Combination of deep eutectic solvent and ionic liquid to improve biocatalytic reduction of 2-octanone with *Acetobacter pasteurianus* GIM1.158 cell

Pei Xu,[1,2] *Peng-Xuan Du,*[2] *Min-Hua Zong,*[1] *Ning Li,*[2] *Wen-Yong Lou*[1,2,*]

[1] State Key Laboratory of Pulp and Paper Engineering, South China University of Technology, Guangzhou 510640, China

[2] Laboratory of Applied Biocatalysis, School of Food Science and Engineering, South China University of Technology, Guangzhou 510640, China

* Corresponding author. Tel.: +86-20-22236669; fax: +86-20-22236669;
E-mail: wylou@scut.edu.cn

Table S1. Partition coefficients of 2-octanone and 2-octanol between two phase systems

Media	Partition coefficients	
	2-octanone	2-octanol
C_4MIM ·PF_6/buffer	129	45
C_5MIM ·PF_6/buffer	115	41
C_2MIM ·Tf_2N/buffer	112	43
C_4MIM ·Tf_2N/buffer	98	39
PP_{14} ·Tf_2N/buffer	86	32

Table S2. Effect of ChCl/EG (1:2) concentration on pH value of the reaction system

DES concentration （v/v，%）	pH
0	5.00
10	4.97
20	4.94
30	4.92
40	4.89
50	4.86
60	4.85

Table S3 Effect of different parts of the cell on the bioreduction of 2-octanone

Catalyst	Coenzyme	Product
Cell extract	no	-
Cell extract	NADH	+
Cell debris	no	-
Cell debris	NADH	-

-: no product detected

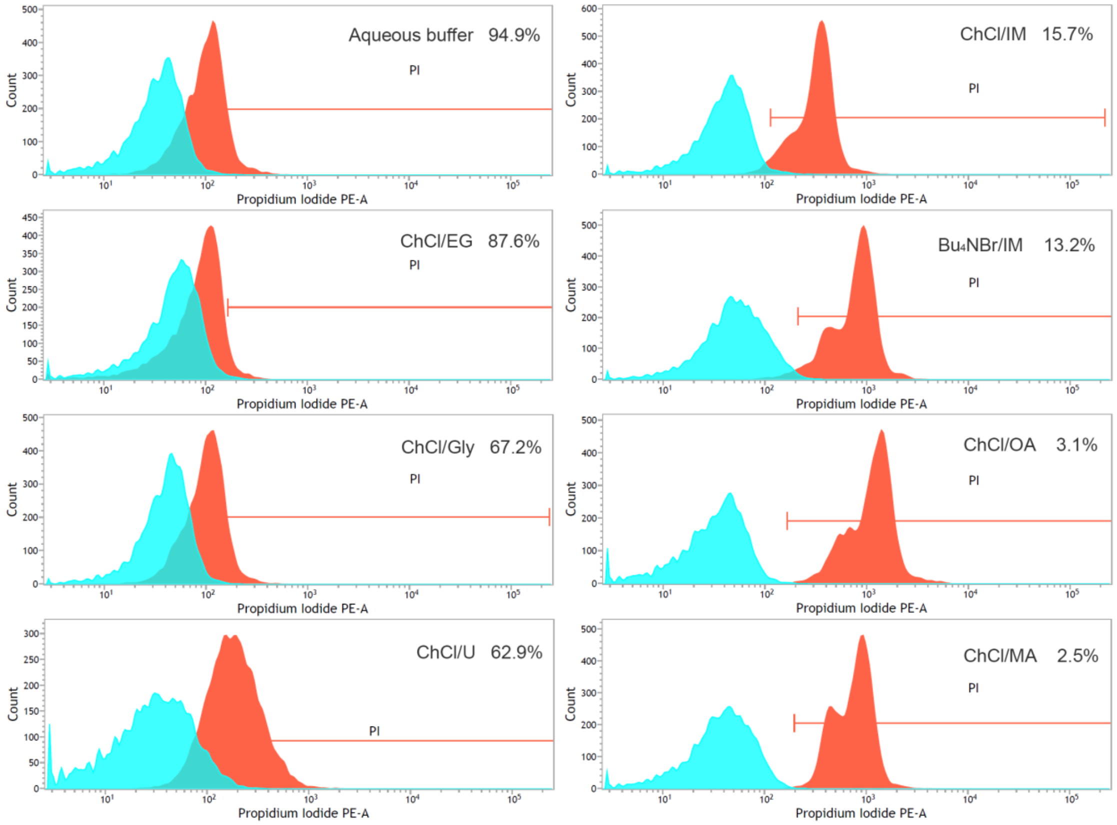

Figure S1. Membrane integrity of *Acetobacter pasteurianus* GIM1.158 cells affected by various DESs

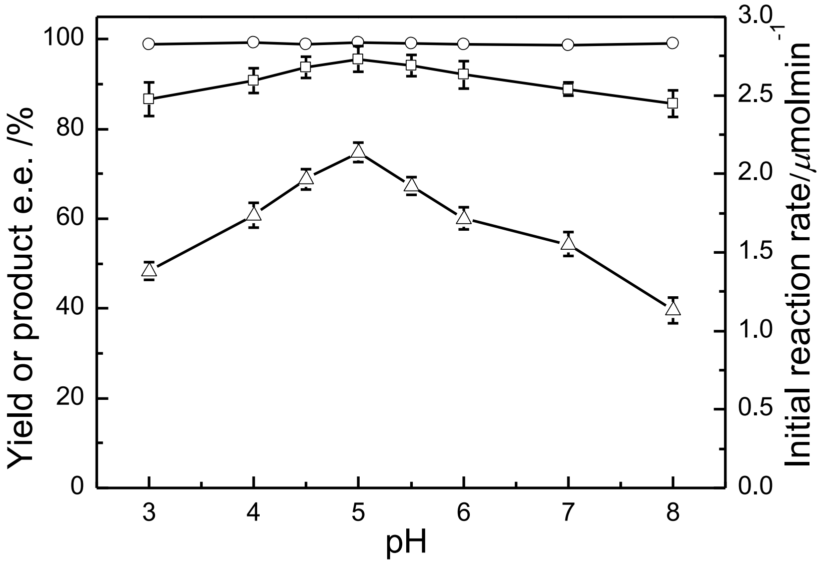

Figure S2. Effect of buffer pH on the product yield (□), product *e.e.* (○) and initial reaction rate (△) of the biosynthesis of (*R*)-2-octanol with the biocatalyst in momophasic system. Reaction condition: TEA-HCl buffer (10 mL, 50 mM, different pHs) containing of ChCl/EG (40% v/v), 2-octanone (40 mM), isopropanol (500 mM), and *Acetobacter pasteurianus* GIM1.158 cell (25 mgmL^{-1}), 35 ℃, 180 rpm.

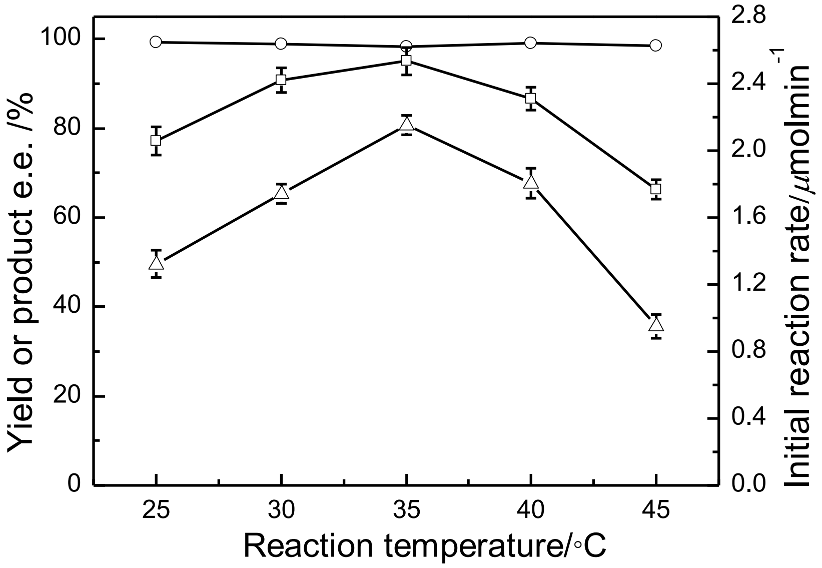

Figure S3. Effect of reaction temperature on the product yield (□), product *e.e.* (○) and initial reaction rate (△) of the biosynthesis of (*R*)-2-octanol with the biocatalyst in momophasic system. Reaction condition: TEA-HCl buffer (10 mL, 50 mM, pH5.0) containing of ChCl/EG (40% v/v), 2-octanone (40 mM), isopropanol (500 mM), and *Acetobacter pasteurianus* GIM1.158 cell (25 mgmL^{-1}), different temperatures, 180 rpm.

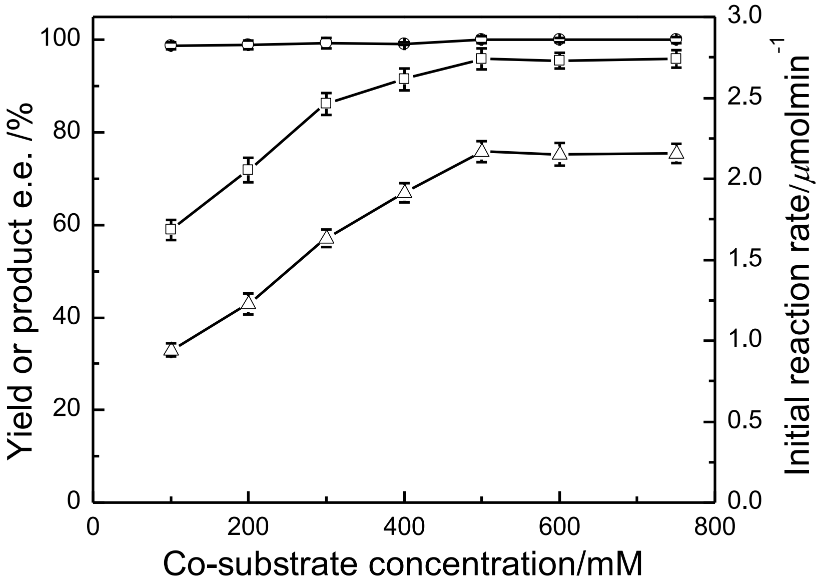

Figure S4. Effect of co-substrate concentration on the product yield (□), product *e.e.* (○) and initial reaction rate (△) of the biosynthesis of (*R*)-2-octanol with the biocatalyst in momophasic system. Reaction condition: TEA-HCl buffer (10 mL, 50 mM, pH5.0) containing of ChCl/EG (40% v/v), 2-octanone (40 mM), different concentrations of isopropanol, *Acetobacter pasteurianus* GIM1.158 cell (25 mgmL^{-1}), 35 ℃, 180 rpm.

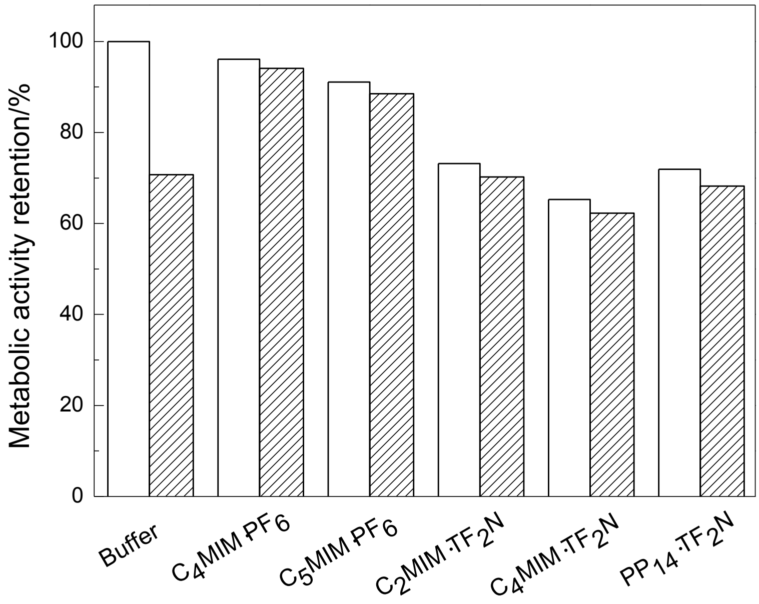

Figure S5. Effect of various ILs on the sugar metabolic activity retention of *Acetobacter pasteurianus* GIM1.158 cells without (white) and with (pattern) substrate.

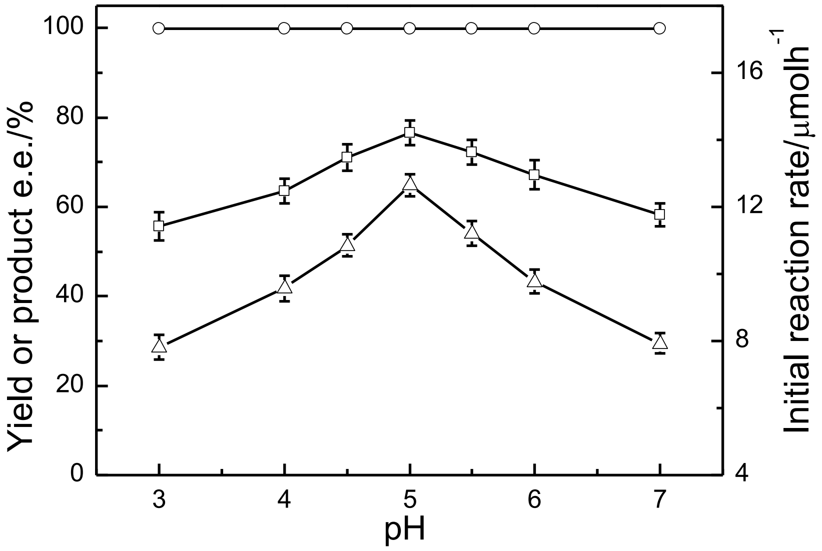

Figure S6. Effect of pH on the product yield (□), product *e.e.* (○) and initial reaction rate (△) of the biosynthesis of (*R*)-2-octanol with the biocatalyst in biphasic system. Reaction conditions: 2-octanone (60 mM), $C_4MIM·PF_6$ (1.0 mL), TEA-HCl buffer (50 mM, different pH,4 mL) containing ChCl/EG (40% v/v), isopropanol (500 mM), *Acetobacter pasteurianus* GIM1.158 cell (25 $mgmL^{-1}$), 35 ℃, 180 rpm.

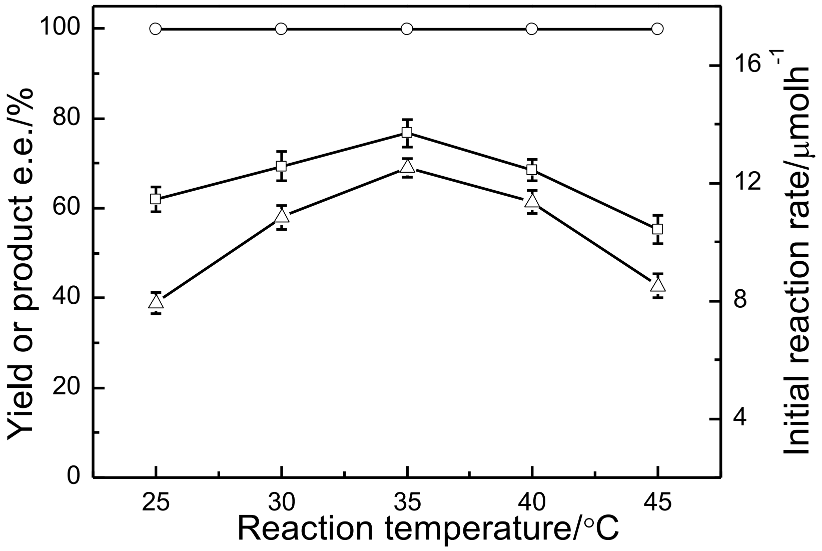

Figure S7. Effect of reaction temperature on the product yield (□), product *e.e.* (○) and initial reaction rate (△) of the biosynthesis of (*R*)-2-octanol with the biocatalyst in biphasic system. Reaction conditions: 2-octanone (60 mM), $C_4MIM·PF_6$ (1.0 mL), TEA-HCl buffer (50 mM, different pH, 4 mL) containing ChCl/EG (40% v/v), isopropanol (500 mM), *Acetobacter pasteurianus* GIM1.158 cell (25 $mgmL^{-1}$),, different temperature, 180 rpm.

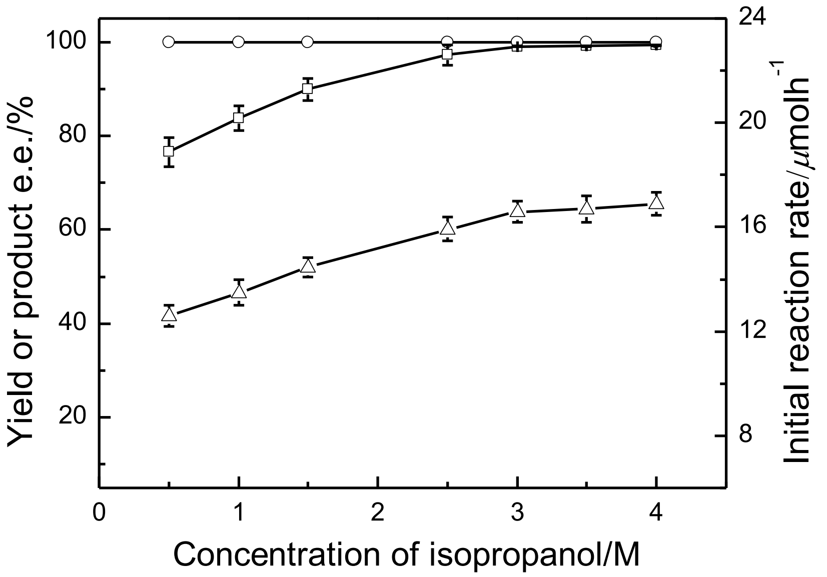

Figure S8. Effect of isopropanol concentration on the product yield (□), product *e.e.* (○) and initial reaction rate (△) of the biosynthesis of (*R*)-2-octanol with the biocatalyst in biphasic system. Reaction conditions: 2-octanone (60 mM), $C_4MIM \cdot PF_6$ (1.0 mL), TEA-HCl buffer (50 mM, different pH, 4 mL) containing ChCl/EG (40% v/v), different concentration of isopropanol, *Acetobacter pasteurianus* GIM1.158 cell (25 mgmL^{-1}), different temperature, 180 rpm.

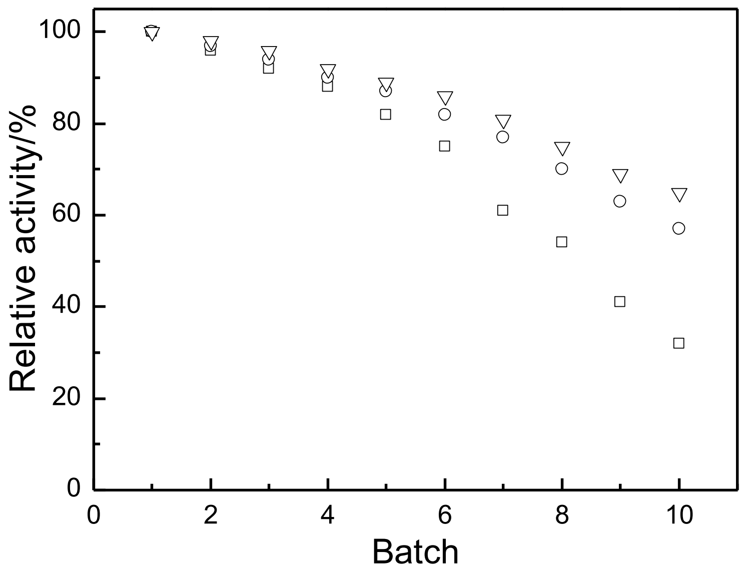

Figure S9. Operational stability of *Acetobacter pasteurianus* GIM1.158 cells in buffer system (□), ChCl/EG-buffer system (○) and the biphasic system (△). Reaction conditions:

Buffer system: TEA-HCl buffer (50 mM, pH5.0, 10 mL) , 2-octanone (40 mM), *Acetobacter pasteurianus* GIM1.158 cell (25 mgmL^{-1}),isopropanol (500 mM), 35 ℃, 120 rpm.

ChCl/EG-buffer system: ChCl/EG-containing (40%, v/v) TEA-HCl buffer (50 mM,

pH5.0, 10 mL) , 2-octanone (60 mM), *Acetobacter pasteurianus* GIM1.158 cell (25 $mgmL^{-1}$), isopropanol (500 mM), 35 ℃, 180 rpm.

ChCl/EG-buffer/ $C_4MIM \cdot PF_6$ system: 2-octanone (1.5 M), $C_4MIM \cdot PF_6$ (1.0 mL), TEA-HCl buffer (50 mM, pH5.0, 4 mL) containing ChCl/EG (40%, v/v), isopropanol (3 M),*Acetobacter pasteurianus* GIM1.158 cell (25 $mgmL^{-1}$), 35 ℃, 180 rpm.
